# A novel model of liver cancer stem cells developed from induced pluripotent stem cells

**DOI:** 10.1038/s41416-020-0792-z

**Published:** 2020-03-17

**Authors:** Said M. Afify, Anna Sanchez Calle, Ghmkin Hassan, Kazuki Kumon, Hend M. Nawara, Maram H. Zahra, Hager M. Mansour, Apriliana Cahya Khayrani, Md Jahangir Alam, Juan Du, Akimasa Seno, Yoshiaki Iwasaki, Masaharu Seno

**Affiliations:** 1 Department of Medical Bioengineering, Graduate School of Natural Science and Technology, Okayama University, Okayama, 700-8530 Japan; 2 Division of Biochemistry, Chemistry Department, Faculty of Science, Menoufia University, Shebin ElKoum Menoufia, 32511 Egypt; 3 Division of Molecular and Cellular Medicine, National Cancer Center Research Institute, Tokyo, 104- 0045 Japan; 4 Laboratory of Nano-Biotechnology, Graduate School of Interdisciplinary Science and Engineering in Health Systems, Okayama University, Okayama, 700-8530 Japan; 5 Department of Microbiology and Biochemistry, Faculty of Pharmacy, Damascus University, Damascus, 10769 Syria; 6 Okayama University Research Laboratory of Stem Cell Engineering in Detroit, IBio, Wayne State University, Detroit, MI 48202 USA; 7 Department of Gastroenterology and Hepatology, Graduate School of Medicine, Okayama University, Okayama, 700-8558 Japan

**Keywords:** Cancer stem cells, Cancer models

## Abstract

**Background:**

Liver cancer is the second most common cause of cancer-related death. Every type of tumours including liver cancer contains cancer stem cells (CSCs). To date, the molecular mechanism regulating the development of liver CSCs remains unknown.

**Methods:**

In this study, we tried to generate a new model of liver CSCs by converting mouse induced pluripotent stem cells (miPSCs) with hepatocellular carcinoma (HCC) cell line Huh7 cells conditioned medium (CM). miPSCs treated with CM were injected into the liver of BALB/c nude mice. The developed tumours were then excised and analysed.

**Results:**

The primary cultured cells from the malignant tumour possessed self-renewal capacity, differentiation potential and tumorigenicity in vivo, which were found rich in liver cancer-associated markers as well as CSC markers.

**Conclusions:**

We established a model of liver CSCs converting from miPS and showed different stages of stemness during conversion process. Our CSC model will be important to assess the molecular mechanisms necessary to develop liver CSCs and could help in defeating liver cancer.

## Background

According to the World Cancer Report, the incidence of liver cancer was globally 6% and the mortality burden was 9%.^1^ With the number of deaths estimated as 746,000 in 2012, liver cancer is the second leading cause of cancer mortality in the world. The liver cancer in men is described as the fifth most common cancer (554,000 new cases, 8% of the total) and that in women the ninth (228,000 cases, 3% of the total). Among the primary liver cancers, hepatocellular carcinoma (HCC) is the major histological subtype.^2^ Hepatocarcinogenesis could be explained by a complexed multistep process at molecular level giving various diagnostic observations in cells and histology. Although the molecular mechanism of the liver cancer development has been studied for many years, these studies focussed only on the cancer cells, which are present in the cancer tissues, but not the origin of these cancer cells, which are known as the liver cancer stem cells (CSCs). Liver CSCs are described with the capacity of self-renewal and differentiation potential.^3^ Liver CSCs are currently considered as a specific subpopulation with significant tumorigenic potential, which should contribute to the development and recurrence of HCC.^4^ Taking the presence of original cells as granted, we support the idea that the liver CSCs could be originated by the transformation of liver stem/progenitor cells.^5^ Actually, liver CSCs are identified by self-renewal and pluripotency and classified with normal liver stem cell markers.

Generally, CSCs are defined by self-renewal, pluripotency and tumorigenicity, which play a critical role in the growth of primary tumours with heterogeneity.^6^ Considering that CSCs are responsible for the malignant tumorigenic potential providing the heterogeneity,^7^ CSCs could be the cells at the top of the hierarchy undergoing differentiation into cancer cells with diverse phenotypes with limited proliferative potential in many cancers as found in the hierarchy of normal stem cells in normal tissues.

Incredible efforts have been made to understand where the CSCs come from. Owing to the recent rapid progress in the stem cell research, cancer is widely accepted as a stem cell disease.^8^ Also, some scientists suggested that hierarchically organised tumours originated from normal stem cells,^9^ which opened the possibility of the liver stem cells to be the origin of liver CSCs.^10^ Stem cells were hypothesised to dwell in a specific microenvironment called a “stem cell niche”, which plays an essential role to regulate stem cell maintenance and self-renewal by secreting various factors.^11^ A similar concept of niche also is considered present and applies to CSCs which is the so called “cancer stem cell niche (CSCN)”, and the interactions of CSCs with this niche should be essential to maintain the CSC population.^12^ Cells within the CSCN secrete factors, which stimulate CSC self-renewal, induce the differentiation such as angiogenesis^13^ and recruit immune cells and other stromal cells, which secrete additional factors to promote tumour cell invasion and metastasis.^14^ The niche for liver CSCs has not yet been elucidated and still obscure, but the mechanisms similar to those of the niche of the normal stem cells should exist to control cell proliferation, migration, invasion and apoptosis resistance.^15^

Recently, stem cells, including embryonic stem cells (ESCs) or induced pluripotent stem cells (iPSCs), have gathered great attention in the field of medicine because of the development of novel therapy of tissue regeneration. On the other hand, the development of CSCs or cancer cells could be possible when normal stem cells are affected by the tumour microenvironment, although the mechanism of development is not clear yet. Our group hypothesises that the CSCs may appear from the normal stem cells affected by the cancer-inducing niche defined as chronic inflammation.^16^ This mechanistic insight, which converts stem cells into liver CSCs, will significantly be important to uncover the molecular mechanisms lying in liver CSC development. In the present study, we tried to develop liver CSCs converting from miPSCs using the conditioned medium (CM) of HCC cell lines mimicking chronic liver disease.

## Methods

### Cell culture

Human HCC cell line Huh7 (Riken Cell Bank, Japan) was cultured in Dulbecco’s Modified Eagle’s Medium (DMEM, Sigma-Aldrich, USA) supplied with 10% foetal bovine serum (FBS) and 100 U/mL penicillin/streptomycin (Wako, Japan). Then cells were incubated in a 37 °C incubator with 5% CO_2_. Medium was changed at 80% confluence to 5% FBS. Culture supernatant, which is known as CM, was collected after 48 h and centrifuged for 10 min at 1000 rpm at room temperature and then filtered through a 0.22 μm filter (Millipore, Ireland). Then 3 mL CM were added into 3.5 cm dish overnight to confirm that there were no surviving cancer cells in CM. Mouse iPSCs (miPSCs) were maintained under the humidified 5% CO_2_ atmosphere at 37 °C on feeder layer of mitomycin-C-treated mouse embryonic fibroblasts (Reprocell, Japan) in miPS medium (DMEM containing 15% FBS, 0.1 mM non-essential amino acid (NEAA), Thermo Fisher Scientific, USA), 2 mM L-Glutamine (Nacalai Tesque, Japan), 0.1 mM 2-mercaptoethanol (Sigma-Aldrich, USA), 1000 U/mL leukaemia inhibitory factor (LIF) (Merck Millipore, USA) and 100 U/mL penicillin/streptomycin (Wako, Japan). After 1 week, miPSCs were transferred to gelatine (Sigma-Aldrich, USA) coated dishes. After 70% confluence, miPSC conversion was started using 1:1 ratio of miPS medium and CM from Huh7 cells for 4 weeks. The converted cells established from miPSCs in the CM for 4 weeks were named as miPS-Huh7cm cells. Using this method, we made three independent miPS-Huh7cm cell lines. Nanog-GFP reporter expression was used in miPSCs and the expression of green fluorescent protein (GFP) reflects the maintenance of stemness. For primary culture, tumour tissues were cut into small pieces in Hank’s Balanced Salt Solution (HBSS). Then these were suspended into a 15-ml tube containing 50% HBSS and 50% of dissociation buffer (0.25% trypsin, 0.1% collagenase, 20% KnockOut™ Serum Replacement (Gibco, NY, USA), 1 mM of CaCl_2_ in phosphate-buffered saline (PBS)) and incubated at 37 °C for 1 h. After digestion, the suspension was mixed well using 1 mL pipette and then waited for 5 min until large pieces settled down. The supernatant was transferred to a new tube containing 1 mL 10% FBS medium to stop digestion. The cellular suspension was centrifuged at 300 rpm for 3 min, and then the supernatant was transferred again to a new tube that was centrifuged at 1000 rpm for 10 min. The cell pellet was then placed in an appropriate volume of miPS medium without LIF, and the cells were seeded into a dish at a density of 1 × 10^5^/mL. These primary cells were named as miPS-Huh7cmP1 cells. Cell morphology was observed and photographed using Olympus IX81 microscope equipped with a light fluorescence device (Olympus, Japan).

### Tumorigenicity assay in vivo

To explore the tumorigenic capacity, mice were euthanised with 2% isoflurane through inhalation and liver orthotopic injections were performed into 4-week-old Balb/c-nu/nu female immunodeficient mice (3 mice for each cell line; Charles River, Japan) with 0.5 × 10^6^ cells suspended in 50 μL HBSS (Gibco, Japan). Tumour formation was monitored weekly after implantation. After 4 weeks, animals were sacrificed by euthanasia with 5% of isoflurane through inhalation to ensure rapid loss of consciousness and respiratory and cardiac arrest followed by cervical dislocation to ensure the death of mice. To investigate the metastatic potential of our novel cells, tail vein injection was performed into 4-week-old Balb/c-nu/nu female immunodeficient mice (*n* = 3; Charles River, Japan) with 5 × 10^6^ cells suspended in 100 μL HBSS (Gibco, Japan). Mice were monitored for up to 6 weeks. Animals were sacrificed after 6 weeks. The plan of animal experiments was reviewed and approved by the ethics committee for animal experiments of Okayama University under the IDs OKU-2013252 and OKU-2016078. All animal experiments have been performed in accordance with the ARRIVE/NC3R guidelines.

### Histological analysis

#### Haematoxylin and eosin staining

Tumours were fixed in 10% formalin (Wako Japan), embedded in paraffin wax and sectioned for histologic examination at 5 μm. Sections were stained with haematoxylin 0.5% (Sigma-Aldrich, USA) and eosin Y (Sigma-Aldrich, USA). Slides were analysed using a light microscope (Eclipse Ti, Japan).

#### Immunohistochemistry (IHC)

IHC performed was the same as standard procedures using the ABC reagent (Vector Laboratories, USA). Detection was accomplished using DAB substrates (Vector Laboratories, USA). Incubation of sections with PBS served as negative controls. Sections were lightly counterstained with haematoxylin and mounted with Micromount (Leica Camera AG, Wetzlar, Germany).

### Quantitative reverse transcription PCR (RT-qPCR)

The total RNA was isolated from the cells or tissues using TRIzol (Life technologies, USA), according to the manufacturer’s protocol. The extracted RNA was treated with DNase I (Promega, USA). One μg of RNA was reverse transcribed using GoScript™ Reverse Transcription System (Promega, USA). RT-qPCR assays were done by Light Cycler 480 II using Light Cycler 480 SYBR green I Master Mix (Roche Diagnostics GmbH, Germany) according to the manufacturer’s instructions. Gene expression level was normalised to that of glyceraldehyde-3-phosphate dehydrogenase mRNA. The primers used for the RT-qPCR analysis are listed in Supplementary Table S1.

### RNA-seq library construction and sequencing

Isolation of total RNA was performed using TRIzol (Life technologies, USA). RNA samples were prepared using Illumina TruSeq RNA Sample Preparation Kit and Illumina HiSeq 2500 was used to sequence samples. Bioinformatics analysis was carried out by Fligen, INC. (Novogene, Nagoya Japan).

### Flow cytometry

Cells were incubated in PBS containing 10% FBS with either fluorescence-conjugated primary antibody or primary antibody followed by secondary antibody (Table S2). Cells were washed and resuspended in PBS containing 10% FBS and analysed by BD AccuriTM C6 plus flow cytometer (Becton & Dickinson, USA). The data were analysed using the FlowJo® software (FlowJo, LLC, Ashland, OR, USA).

### Sphere-formation assay

For spheroids initiated with CSCs, serum-free medium, DMEM, supplied with NEAA (1%), L-glutamine (1%), 100× penicillin/streptomycin (0.5%), β-mercaptoethanol (β-ME; 0.1 mM) and Insulin-Transferrin-Selenium-Ethanolamine (ITS-x) (1/100 v/v) were used for hanging drop method. Cells were suspended in medium to get cell density of 2.5 × 10^4^ cells/mL. Each hanging drop contained 20 μL of volume, and the bottom dish contained 10 mL PBS. Dishes were incubated at 37 °C in 5% CO_2_ for 3 days, then formed spheres were transferred to non-coated dish and analysed.

### Limiting dilution assay

Cells were washed and subjected to enzymatic dissociation. To investigate the percentage of single cells capable of forming new spheres, cells were resuspended in serum-free medium supplied with serum-free medium, DMEM, supplied with NEAA (1%), L-glutamine (1%), 100× penicillin/streptomycin (0.5%), β-ME (0.1 mM) and ITS-x (1/100 v/v) and seeded at dilution 500, 200, 100, 10 and 1 cell in 96-well low-attachment plates (EZ Bind Shut TMSP, Japan). After 7 days of stem cell incubation, the frequency was calculated with the software available at http://bioinf.wehi.edu.au/software/elda/index.html.

### In vitro tube-formation assay

Cells, 5 × 10^5^ cells, were collected, resuspended in endothelial basal medium EBM2 media (EBM-2 Single Quots Kit, Lonza, Switzerland) and seeded in 12-well plates coated with growth factor-reduced Matrigel (Corning Inc., Corning, USA) for 24 h in the presence or absence of angiogenic factors (human epidermal growth factor (5 ng/mL), vascular endothelial growth factor (VEGF; 0.5 ng/mL), R3-insulin-like growth factor-1 (20 ng/mL), ascorbic acid (1 µg/mL), hydrocortisone (0.2 µg/mL), human basic fibroblast growth factor (FGF; 10 ng/mL), heparin (22.5 µg/mL) and FBS (0.02 mL/mL)). Experiments were performed in triplicate. Images of formed tubes were captured by Olympus IX81 microscope (Olympus, Japan).

### Immunofluorescence

For immunofluorescence analysis, after incubation, cells were washed with PBS, fixed with 4% paraformaldehyde for 20 min at room temperature and subsequently permeabilised with 100% methanol. The cells were incubated in blocking solution (PBS supplemented with 10% FBS) for 1 h followed by incubation overnight at 4 °C with the primary antibody (Table S2). Then cells were washed and incubated with the secondary antibody. After removal and proper washing of secondary antibody, nuclei were counterstained with 4, 6-diamino-3-phenylidole dihydrochloride (Sigma-Aldrich, USA) and mounted on glass sides using Vecta-shield mounting medium (Vector Labs, USA). Images were taken by Olympus IX81 inverted microscope.

### Scratch wound-healing assay

Cells were seeded in 60-mm dishes at 5 × 10^5^ cells/dish and incubated for a 24-h period in miPS media to allow formation of a confluent monolayer. The miPS media was removed and the confluent cell sheet was wounded through scratching the culture well surface with a 200 μL pipette tip. The scratch-wounded cells were washed three times with PBS to remove any cell fragments or detached cells before incubating in fresh media for 48 h. Cell migration was monitored, and images of wound healing were captured by using the microscope after 24 and 48 h.

### Cell invasion assay

Cell invasion potential was evaluated using a Corning Matrigel Invasion Chamber (Corning Inc., USA), which consisted of a Matrigel-coated transwell and transwell inserts. First of all, inserts were coated with ice-cold growth factor-reduced Matrigel and incubated at 37 °C for at least 2 h. Then 5 × 10^4^ cells were suspended in 500 μL serum-free medium, DMEM, seeded onto the insert, and 750 μL medium supplemented with 15% FBS was added to the lower chamber. After incubation for 72 h in 5% CO_2_ atmosphere at 37 °C, non-invasive cells were removed by wiping, and cells that had invaded the Matrigel were fixed in 4% paraformaldehyde (Nacalai Tesque, Japan) for 5 min and subsequently fixed in methanol (Wako, Japan) for 20 min. Cells were stained with Azure EMB Giemsa (Merck Millipore, USA) and quantitatively analysed under a light microscope.

### Statistical analysis

Statistical analyses were performed with Prism 7 (Graph Pad Software, USA) using one-way analysis of variance (ANOVA) followed by Tukey’s multiple comparison test when ANOVA indicated a statistical significance existed with *p* < 0.05 indicating a statistically significant difference. All experiments were summarised as mean ± SD.

## Results

### miPSCs survived in the presence of CM of hepatocyte-derived carcinoma cell line Huh7 cells

Usually, iPSCs are considered to be induced progenitor cells, which differentiate into various normal phenotypes, just like ESCs, depending on the normal niche. On the other hand, cancer-inducing niche could be conceivable as chronic condition. In this study, we tried to differentiate miPSCs into liver CSCs using the CM from HCC cell line Huh7 cells, which exhibited significant expression of liver cancer markers, such as glypican 3, alpha fetoprotein (AFP) and arginase 1 genes, without any genetic manipulation and keeping serial transplantation of primary cultures in the liver. Simultaneously, Huh7 cells were found by gene expression meta-analysis using ExAtlas^17^ to be overexpressing inflammatory-related secretory factors, such as interleukin-18, C-X-C chemokine motif ligands 1, 5, 6, FGF-19, bone morphogenetic protein 2 and thrombospondin 4 when compared with PLC/PRF/5 and/or Hep G2 cells (Supplementary Fig. 1). We supposed that the CM from Huh7 cells could be available to mimic chronic inflammation as the microenvironment of miPSCs. As the result, miPSCs survived in the presence of CM of Huh7 cells instead of LIF for 4 weeks, while miPSCs cultured without LIF differentiated and stopped growing by losing GFP expression during the first week (Supplementary Fig. 2). The survived miPSCs treated with CM were named miPS-Huh7cm cells.

### miPS-Huh7cm cells exhibited high tumorigenicity with liver CSC signature

**Fig. 1:**
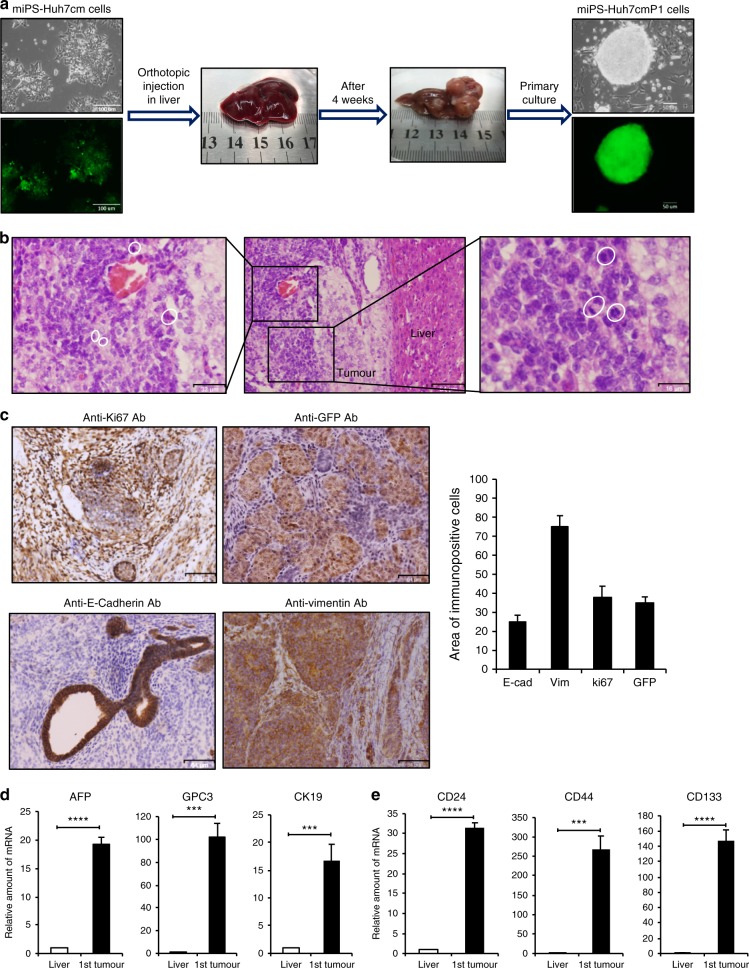
Tumorigenicity of miPSCs treated with the conditioned medium of Huh7 cells. **a** Representative scheme of the orthotopic injection of miPS-Huh7cm cells (miPS treated with Huh7cm for 4 weeks) into the liver and the formed tumour after 4 weeks of injection. Scale bars represent 100 and 50 µm. **b** Histopathological features of the primary tumours were evaluated by H&E staining, showing the presence of mitotic figures (white cycle). Scale bars represent 64, 32 and 16 µm. **c** Immunostaining of the malignant tumours for Anti-ki67 Ab, Anti-GFP Ab, Anti-E-Cadherin Ab and Anti-Vimentin Ab; and histogram showing area of immunopositive cells of each marker. All data were from three independent experiments (*n* = 3). Scale bars represent 64 μm. **d** RT-qPCR analysis for liver cancer-associated biomarkers in the derived tumour tissue compared to liver. All data were from three independent experiments (*n* = 3) (***p < 0.0001, ****p < 0.00001). **e** RT-qPCR analysis of CSC markers in the derived tumour tissue compared to liver. All data were from three independent experiments (*n* = 3) (***p < 0.0001, ****p < 0.00001).

To evaluate the ability to form malignant tumours in vivo, 5 × 10^5^ of miPS-Huh7cm cells were injected into the liver. miPS-Huh7cm cells gave rise to 9 malignant tumours out of 9 mice after 28 days of injection (Fig. 1a), while untreated miPSCs gave rise teratoma-like phenotype with various germ layers (Supplementary Fig. 3).

Tumours were excised and partly cultured on gelatine-coated dish in miPS medium to obtain the primary culture. From the primary culture, which exhibited GFP-expressing colonies surrounded by the progenies of myofibroblast-like cells, GFP expressing puromycin-resistant cells were selected and named as miPS-Huh7cmP1 cells (Fig. 1a).

Histology of miPS-Huh7cm cell-derived tumours showed that a substantial portion of the tumours expressed a malignant phenotype such as high nuclear-to-cytoplasmic ratio and mitotic figures (Fig. 1b). Simultaneously, IHC of the tumours showed high Ki-67 expression, which indicated high proliferation rate, and GFP expression, which indicated undifferentiated cell population derived from miPSCs (Fig. 1c). IHC also showed positive staining for E-cadherin and Vimentin implying the presence of heterogeneous cell phenotypes in the miPS-Huh7cm cell-derived malignant tumours. Protein expression was quantitated using the Image J software for those markers and showed that approximately 30% of tumour sections expressed Ki67 and GFP while Vimentin was 70% and E-Cadherin was 20%.

AFP, GPC-3 and CK19 are considered as biomarkers of malignant liver tumour^18–20^ and the combination of these markers improves the accuracy of diagnosis. Using RT-qPCR analysis, we found statistically significant overexpression of these biomarkers in miPS-Huh7cm cell-derived tumour tissues. The relative amount of mRNA expression was enhanced by 16-, 18- and 100-folds for CK19, AFP and GPC3 genes, respectively, when compared to normal mice liver as control (Fig. 1d). Simultaneously, the expression of CSC markers in the tumour tissues were enhanced by 30-, 150- 200-folds for CD24, CD133 and CD44, respectively, when compared to miPSCs (Fig. 1e).

**Fig. 2:**
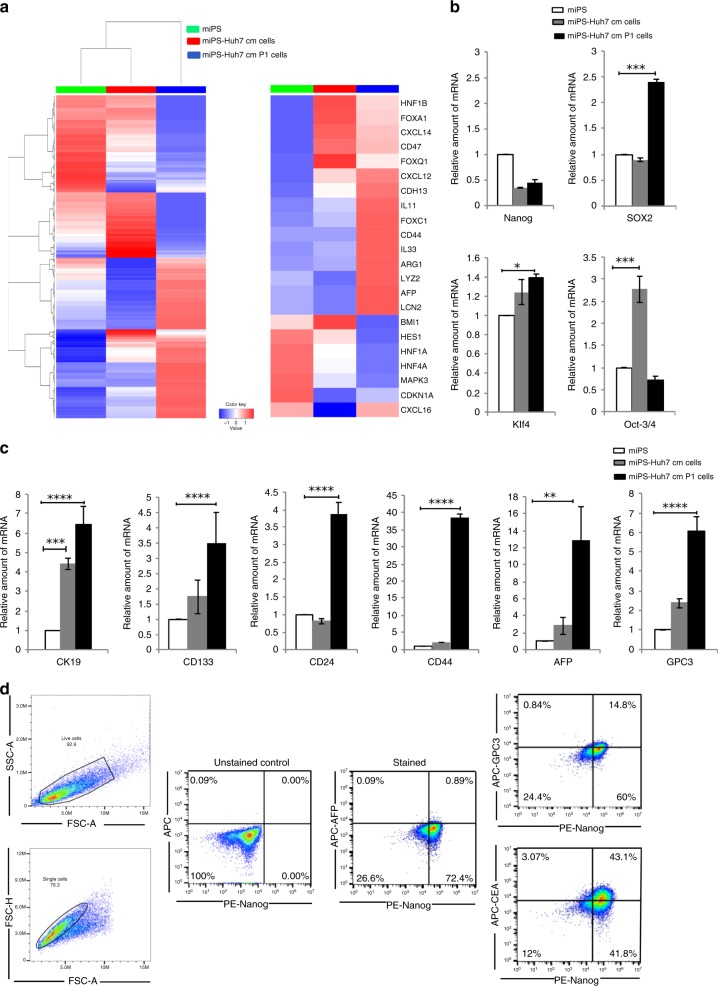
Characterisation of the primary culture of the tumour cells derived from miPS-Huh7cm cells injected into the liver. **a** Representative cluster analysis among miPS, miPS-Huh7cm cells and miPS-Huh7cm P cells (left). The colour range represents the log10(FPKM+1) value from large (red) to small (blue). Main oncogenes and CSC markers implicated in liver cancer are enlarged (right). **b** Stemness marker gene mRNA expression levels were analysed through RT-qPCR analysis in miPS-Huh7cm cells, and miPS-Huh7cm P cells were compared to miPS (*p < 0.01, **p < 0.001, ***p < 0.0001, ****p < 0.00001). **c** Liver cancer stem cell marker gene mRNA expression levels were analysed through RT-qPCR analysis in miPS-Huh7cm cells, and miPS-Huh7cm P cells were compared to miPS (**p < 0.001, ***p < 0.0001, ****p < 0.00001). **d** Flow cytometric analysis for liver cancer-associated markers such as GPC3, AFP and CEA with Nanog in miPS-Huh7cmP1 cells. Each result is shown as a representative of at least five independent experiments.

To further confirm the molecular analysis of the CSCs derived from miPSCs, RNA-sequencing was performed. The expression of liver cancer-related markers such as AFP and Arg1 were found to change significantly together with liver progenitor network genes such as Hnf1a, Hnf1b, Foxa1, Hnf4a, Foxc1, Foxc1, Foxq1 and Hes1 as well as main oncogenes and liver CSC markers implicated such as CD44, CD47, Cxcl12, Lcn2, Lyz2, CDKN1A, Mapk3, Cdh13, Cxcl14, Cxcl16, Il11 and Il33. The expression level changes as shown in the heat map (Fig. 2a) suggest the possible roles of those genes in liver CSC development and could explain the functions of those gene in liver cancer progression. Moreover, the expression level of stem cell marker genes such as Nanog, Sox2, Klf-4 and Oct-4 transcription factors in miPS-Huh7cm and miPS-Huh7cmP1 cells showed that both cells sustain stemness property (Fig. 2b).

To further confirm that the miPS-Huh7cmP1 cells were enriched with the cells expressing liver cancer-associated markers (AFP, GPC3 and CK19) and CSC markers (CD44, CD133 and CD24), we examined the gene expression levels compared to those in miPSCs and miPS-Huh7cm cells. Liver cancer-associated and CSC markers, as expected from the results of the analyses of tumour tissue (Fig. 1d), showed significant elevation again in miPS-Huh7cmP1 cells when compared to miPSCs (Fig. 2c).

Using flow cytometry, we further confirmed the expression of liver cancer-associated markers such as AFP, GPC3 and carcinoembryonic antigen (CEA) in miPS-Huh7cmP1 cells in comparison with Nanog (Fig. 2d). AFP was detected in only 1% of the cells suggesting that the cells are still undifferentiated as liver cancer cells independent of Nanog expression. More than 40% of the cells were CEA^+^/Nanog^+^ suggesting that CEA could be a marker of early stage of differentiation. In contrast with these two markers, GPC3 expression does not appear correlated with Nanog expression. In some populations (24%), GPC3 expression was not showing up with the decrease of Nanog expression while in other population (15%) GPC3 expression showed up while Nanog was still positive (Fig. 2d). Collectively, GPC3 expression should be an intermediate marker among the three markers.

### Tumorigenic potential of miPS-Huh7cm cells was kept during serial orthotopic transplantation

To confirm that miPS-Huh7cm cells maintained tumorigenic potential, serial transplantations of the primary cultures were evaluated for tumour formation. The secondary tumour was obtained by orthotopically injecting 5 × 10^5^ miPS-Huh7cmP1 cells, which were the primary cells from the miPS-Huh7cm-derived tumour, into the liver **(**Supplementary Fig. 4a). Similarly, the third tumour was obtained by injecting 5 × 10^5^ miPS-Huh7cmP2 cells to get primary cells of miPS-Huh7cmP3 cells. Simultaneously, tumour-derived cells were maintained by orthotopically transplanting tumour tissue minced into 1–3 mm diameter.

The tumours developed by the second and the third injection spread in different lobes of the liver even only after 3 weeks of injection. Existed tumours exhibited the phenotypes of malignancy such as multiple pathological mitotic figures, glandular epithelial hyperplasia, varying cell sizes, irregular cell forms, high nuclear-to-cytoplasmic ratio and severe nuclear atypia (Supplementary Fig. 4b). From these results, we concluded that the orthotopic malignant liver cancer model was successfully established with the injection of miPS-Huh7cm cells and the primary cultured cells (miPS-Huh7cmP1 and miPS-Huh7cmP2).

### The liver CSC signatures were enhanced under liver environment in vivo

**Fig. 3:**
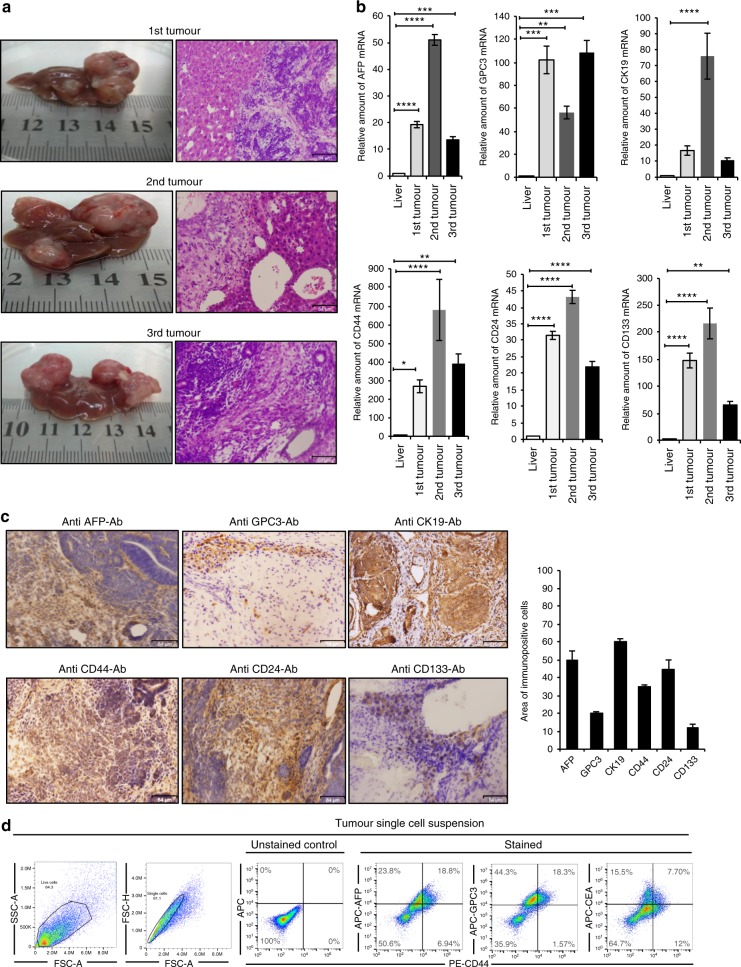
The liver CSC signatures were enhanced in vivo. **a** Representative images of tumour and H&E staining for first, second and third primary orthotopic injection tumour sections. Scale bars represent 64 μm. **b** RT-qPCR analysis of AFP, GPC3 and CK19 were examined and liver CSC markers such as CD133, CD44 and CD24 for the three malignant tumour tissues were compared to those in normal mice liver. Gene expression levels were normalised to those of GAPDH (*p < 0.01, **p < 0.001, ***p < 0.0001, ****p < 0.00001). **c** Immunohistochemical staining of formalin-fixed paraffin-embedded liver tumour tissue derived from serial transplantation (miPS-Huh7cm cells) and histogram showing area of immunopositive cells of each marker. Samples representative of each tumour were stained with antibodies recognising AFP, GPC3 and CK19. Also, antibodies recognising liver CSC like CD44, CD24 and CD133 were applied to the tumour tissues. All data were from three independent experiments (*n* = 3). Scale bars represent 64 μm. **d** FACS analysis shows AFP^+^/CD44^+^, GPC3^+^/CD44^+^ and CEA^+^/CD44^+^ subpopulations in the tumour single-cell suspension.

Liver CSC signatures were evaluated to be maintained in miPS-Huh7cm cells keeping tumorigenic potential during the serial transplantation. The malignant tumour tissues from the three injections (Fig. 3a) were further analysed for the liver CSC signature. First, the expression levels of liver cancer-associated markers, AFP, GPC3 and CK19 as well as liver CSC markers, CD44, CD24 and CD133, in malignant tumours were relatively high compared to that in normal liver by RT-qPCR (Fig. 3b). AFP expression increased significantly (*p* ≤ 0.01) by 19-, 50- and 14-folds in first, second and third tumours, respectively, when compared to normal mice liver. GPC3 was found significantly (*p* ≤ 0.01) overexpressed in all tumours by 68-, 50- and 96-folds in first, second and third tumours, respectively. CK19 was also elevated in all tumours by 17-, 76- and 11-folds in first, second and third tumours, respectively.

Second, liver CSC phenotypes were immunohistochemically assessed in the primary tumours derived from miPS-Huh7cm cells injected into the liver. Immuno-reactive liver cancer markers AFP, GPC3 and CK19 were positively stained as well as the CSC markers such as CD44, CD24 and CD133 in the tumour tissue developed in the liver (Fig. 3c). Protein expression was quantitated using the Image J software for those markers and showed that approximately 50% of tumour sections expressed AFP and CK19 while for GPC3 it was 20% and for CD44 it was 30% (Fig. 3c). Finally, single-cell suspension from tumour tissues was subjected to flow cytometry to assess the liver CSC markers, such as AFP, GPC3, CEA and CD44 (Fig. 3d). The result showed the presence of subpopulations that were double positive for AFP^+^/CD44^+^, GPC3^+^/CD44^+^ and CEA^+^/CD44^+^. Among the AFP^+^ subpopulations, two populations of AFP^+^/CD44^−^ (23.8%) and AFP^+^/CD44^+^ (18.8%) were present while AFP^−^/CD44^+^ was found to be 6.94%. As for the GPC3^+^ subpopulations, two populations GPC3^**+**^/CD44^**−**^ (44.3%) and GPC3^+^/CD44^+^ (18.3%) were found. For CEA^+^ subpopulations, there were two subpopulations: one was CEA^**+**^/CD44^−^ (15.5%) and another was CEA^+^/CD44^+^ (7.7%). These results indicate that miPS-Huh7cm cells successfully differentiated to exhibit liver CSC characters after injection into the liver providing malignant tumours with the markers of liver CSCs as well as those well accepted as liver cancer markers.

The primary cultures from serial transplantation exhibited GFP-expressing colonies surrounded by myofibroblast-like cells indicating that serial transplantation maintained the presence of CSCs (Supplementary Fig. 5a). The expression of liver cancer markers was compared between all primary cultured cells derived from the serial transplantation (miPS-Huh7cmP1, miPS-Huh7cmP2 and miPS-Huh7cmP3 cells) and miPS-Huh7cm cells compared to normal liver while the expression of liver CSC markers was compared to miPSCs (Supplementary Fig. 5b). The expression of GPC3 and CK19 was found significantly upregulated in the primary cultured cells when compared to normal liver while the expression of AFP was downregulated. CD44 expression in the primary cultured cells was extremely elevated in miPS-Huh7cmP2 and miPS-Huh7cmP3 cells by 100- and 275-folds, respectively, when compared to miPS. The expression of CD24 and CD133 was significantly elevated at the same time indicating the CSC characters. Collectively, these data suggest that serial transplantation helps maintaining the presence of liver CSCs as the core population in vivo.

### miPS-Huh7cm and miPS-Huh7cmP1 cells exhibited high self-renewal potential

**Fig. 4:**
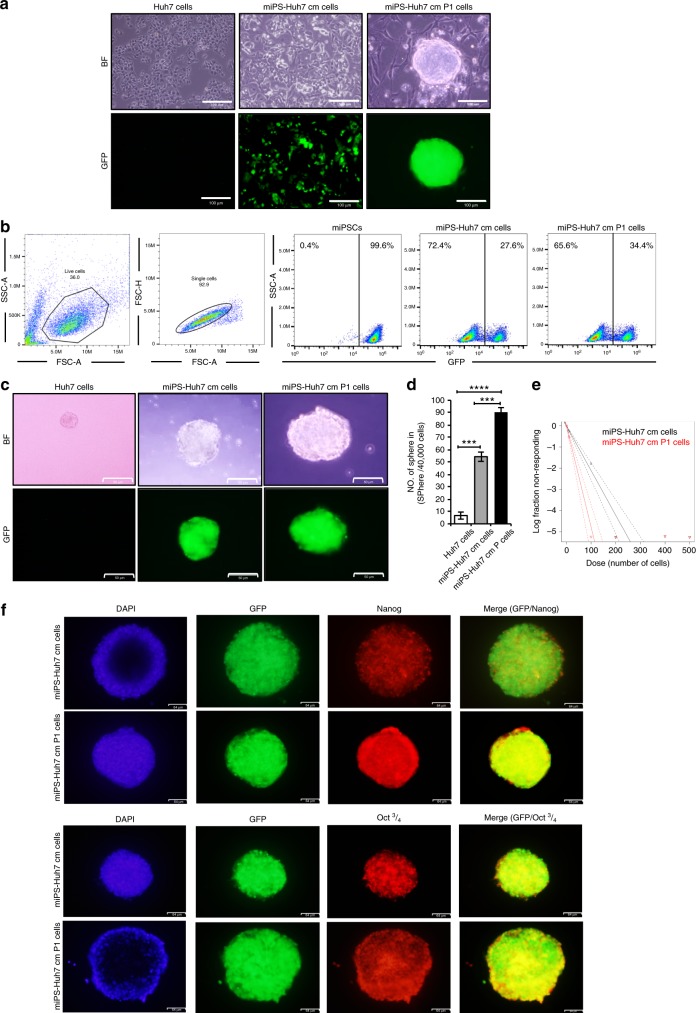
Self-renewal potential of the CSC-like cells. **a** Representative images for adherent culture of Huh7, miPS-Huh7cm and miPS-Huh7cmP1 cells. Scale bars represent 100 μm. **b** FACS analysis shows GFP population in miPS-Huh7cm and miPS-Huh7cmP1 cells. **c** Sphere-formation assay in serum-free medium shows spherogenic potential of both miPS-Huh7cm and miPS-Huh7cmP1 cells compared to Huh7 cells. Scale bars: 50 μm. **d** Number of spheres formed from miPS-Huh7cmP1 cells compared to miPS-Huh7cm and Huh7 cells. **e** Extreme limiting dilution assay assessment of the limiting dilution sphere-forming potential of miPS-Huh7cm and miPS-huh7cmP1 cells. **f** Immunofluorescence staining for Nanog and Oct 3/4 in tumour spheroid derived from miPS-Huh7cm and miPS-Huh7cmP1 cells. Scale bars represent 64 μm.

The self-renewal capacity of miPS-Huh7cm and miPS-Huh7cmP1 cells was assessed for further properties as CSCs. In adhesive culture condition, both cells exhibited two different types of populations; one was colony-expressing GFP and the other was fibroblast-like cells attached to the bottom of dish without expressing GFP (Fig. 4a). The ratio of GFP-positive and -negative cells was estimated by flow cytometry. miPS-Huh7cm and miPS-Huh7cmP1 cells contained GFP-positive cells at 27% and 35%, respectively, while undifferentiated miPSCs were all GFP positive (Fig. 4b). The undifferentiated GFP-positive cells were recognised as sphere-forming population (Fig. 4c), while the GFP-negative fibroblast-like cells could not survive in non-adhesive condition of hanging drop, which would provide a small number of cells such as CSCs with a three-dimensional isolated suspension environment where they were maintained as spheroids.^21^ miPS-Huh7cmP1 cells showed significantly high spheroid-forming potential when compared to miPS-Huh7cm cells or Huh7 cells. Also, the sphere size was larger in miPS-Huh7cm P1 when compared to miPS-Huh7cm as well as Huh7 cells (Fig. 4d). This indicated that both cells had self-renewal capacity. Extreme limiting dilution assay was performed to further confirm the sphere-forming potential when low in number. Results show that miPS-Huh7cmP1 cells exhibited the potential at significantly lower number of cells when compared to that of miPS-Huh7cm cells (Fig. 4e). Spheres derived from miPS-Huh7cm and miPS-Huh7cmP1 cells were assessed for the expression of Nanog and Oct 3/4 since Nanog and Oct 3/4 are considered as critical factors to maintain the undifferentiated state and self-renewal of stem cells.^22^ Both types of cells were found to be positive for both markers (Fig. 4f). Collectively, the self-renewal potential was confirmed in both miPS-Huh7cm and miPS-Huh7cmP1 cells.

### miPS-Huh7cm and primary cultured cells can differentiate into vascular endothelial-like cells

**Fig. 5:**
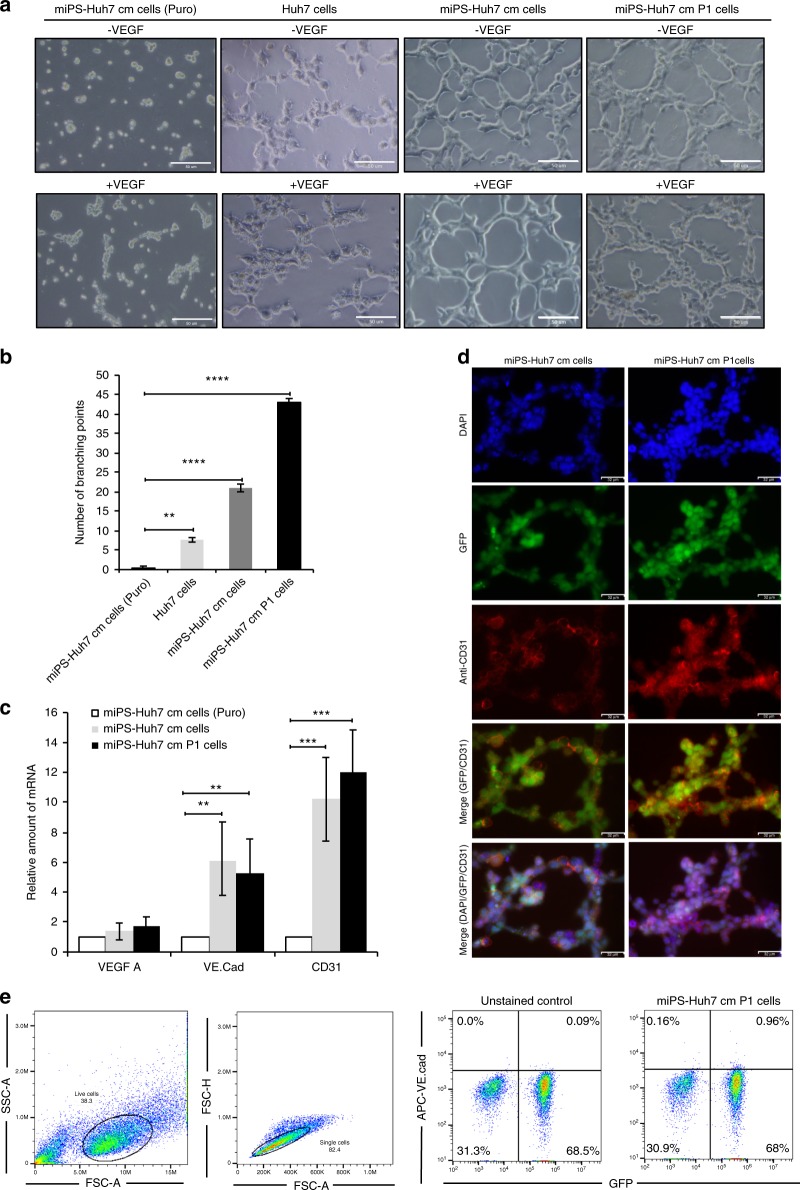
miPS-Huh7cm cells have the potential to differentiate into vascular endothelium-like cells. **a** Tube-formation assays in Huh7, miPS-Huh7cm (Puro), miPS-Huh7cm and miPS-Huh7cmP1 cells after 24 h of culturing on Matrigel. Scale bars represent 50 μm. **b** The number of branching points per field in miPS-Huh7cm and miPS-Huh7cmP1 cells compared to Huh7 and miPS-Huh7cm cells treated with puromycin. **c** The expression levels of VEGF-A VE-cadherin and CD31 were quantified by RT-qPCR. Gene expression levels were normalised to those of GAPDH (**p < 0.001, ***p < 0.0001). **d** Immunofluorescence staining was performed using anti-CD31 antibody in tubular structure derived from miPS-Huh7cm and miPS-Huh7cmP1 cells. Data are the results of three independent experiments. Scale bars represent 50 μm. **e** The population of VE-cadherin-positive cells in primary cells were quantified by flow cytometry. All data were from three independent experiments (*n* = 3). miPS-Huh7cm cells (Puro): miPS-Huh7cm cells treated with puromycin.

Differentiation potential is another property of CSCs as well as self-renewal. miPS-Huh7cm and miPS-Huh7cmP1 cells were assessed for the potential to differentiate into endothelial-like cells forming capillary-like tubes on Matrigel. Formation of capillary-like tubes were confirmed by these cells indicating pro-angiogenic properties of miPS-Huh7cm and miPS-Huh7cmP1 cells in tumorigenesis (Fig. 5a). Both cells showed high potential of tube formation even without VEGF-A when compared to Huh7 or miPS-Huh7cm puromycin-treated cells. miPS-huh7cmP1 cells showed significant elevation in the number of branching points per field when compared to miPS-Huh7cm, Huh7 and miPS-Huh7cm cells treated by puromycin (Fig. 5b).

Since the results implied the possible role of miPS-Huh7cm and miPS-huh7cmP1 cells in tumour angiogenesis, the expression of vasculogenesis-associated factors such as VEGF-A, VE-cadherin and platelet-endothelial cell adhesion molecule-1 (CD31) were evaluated in both cells.^23^ As a result, both cells were found to express VEGF-A but no significant difference was observed between the expression levels in those cells in the adhesive condition, which contained both differentiated and undifferentiated cells, and that in undifferentiated population limited by puromycin. On the other hand, significant difference in the expression of VE-cadherin and CD31 genes were observed between the two different conditions (Fig. 5c). Stained with anti-CD31 antibody, the tubular structure derived from both cells exhibited high expression of CD31 in both cells (Fig. 5d). Flow cytometric analysis revealed the presence of subpopulation positive for VE-cadherin (Fig. 5e). The expression of these angiogenic factors and tube-formation assay results support the differentiation potential of miPS-Huh7cm and miPS-Huh7cmP1 cells. The angiogenic potential of the cells may enhance the concept of angiogenesis, which has been considered to play a pivotal role in tumour growth.^12,13,24^

### miPS-Huh7cm and primary cultured cells exhibited invasion and migration capacity in vitro

**Fig. 6:**
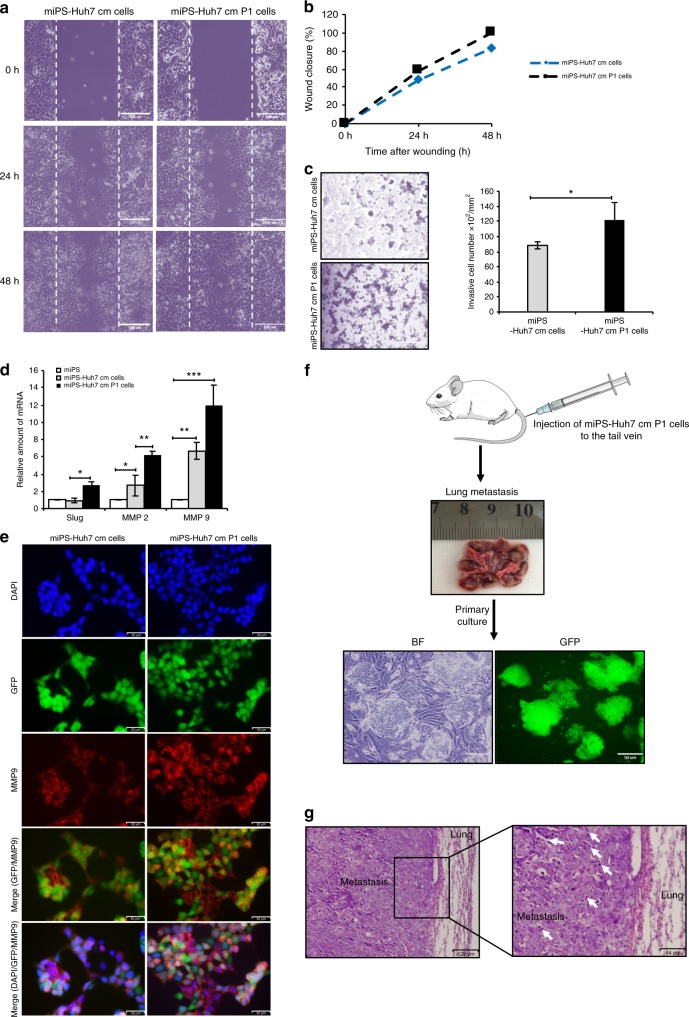
miPS-Huh7cm and primary cells have high metastatic potential. **a** Representative images from wound-healing assay of miPS-Huh7cm and primary cells derived from the primary tumour (miPS-Huh7cmP1 cells). Scale bars represent 100 μm. **b** Summary bar graph illustrating the percentage of wound closure at the indicated time points during the scratch wound assay. **c** Invasion assay performed in miPS-Huh7cm and miPS-Huh7cmP1 cells and summary bar graph illustrating the number of invaded cells on the lower side of the filter. Scale bars represent 100 μm. **d** Expression levels of Slug, MMP2 and MMP9 in both miPS-Huh7cm and miPS-Huh7cmP1 cells compared to miPSCs. Gene expression levels were normalised to those of GAPDH (*p < 0.01, **p < 0.001, ***p < 0.0001). **e** Immunofluorescence staining was performed using anti-MMP9 antibody for miPS-Huh7cm and miPS-Huh7cmP1 cells. Data are the results of three independent experiment. Scale bars represent 32 μm. **f** Metastatic potential of miPS-Huh7cmP1 cells in vivo showing metastasis tumour in the lung and GFP-positive cells isolated from metastatic tumour. Scale bars represent 50 μm. **g** Representative images of haematoxylin–eosin-stained sections of metastatic tumour in the lung show abnormal mitotic figures (white arrow). Scale bars represent 129 and 64 μm.

In the past couple of years, scientists well accepted that CSCs have critical role in metastasis.^25,26^ Steps in the metastatic cascade involve the migration and invasion of the cells degrading the extracellular matrix and cell-to-cell adhesion. The metastatic potential of miPS-Huh7cm and miPS-Huh7cmP1 cells were assessed in vitro by the scratch wound-healing assay for cell migration and by the transmembrane assay for cell invasion. In the scratch wound-healing assay, the motility of miPS-Huh7cmP1 cells was significantly higher than that of miPS-Huh7cm cells (Fig. 6a, b). The percentage of wound closure area after 24 h was 48% and 58% for miPS-Huh7cm and miPS-Huh7cmP1 cells, respectively, while it was 82% and 100%, respectively, after 48 h.

As for the invasive capacity, cells were seeded onto Matrigel-coated transwell membranes and the number of the cells invaded to the other side of the filter was counted after 72 h (Fig. 6c). The ability of invasion was higher in miPS-Huh7cmP1 cells than in miPS-Huh7cm cells. Since Slug, matrix metalloproteinase 2 (MMP2) and MMP9 are considered to be responsible for invasion and migration,^27^ the expression levels of the corresponding genes in miPS-Huh7cm and miPS-Huh7cmP1 cells were assessed and found to be significantly higher than those in miPSCs (Fig. 6d). MMP9 was further confirmed by immunostaining (Fig. 6e). These results suggest that the invasive ability of miPS-Huh7cm cells were enriched during the tumour development in vivo.

### Primary cultured cells exhibited metastatic potential in vivo

Primary culture cells (miPS-Huh7cmP1 cells) produced tumours that grew in the lung after 6 weeks of cell injection into the tail vein. Interestingly, the lungs of the mice with tumours showed redness, swelling and large separated tumours with small parts of normal lung suggesting that most of the lung were invaded by metastasis (Fig. 6f). The primary culture from the metastatic parts showed two different types of populations: one was expressing GFP and the other was negative for GFP confirming that our cells metastasised to the lung from tail vein injection. Indeed, histopathological analysis of representative haematoxylin–eosin-stained sections of metastasis in the lung showed malignant phenotype with abnormal mitotic figures, cytoplasmic degeneration and high nuclear-to-cytoplasmic ratio (Fig. 6g). This result confirms the metastatic potential of our novel cells.

## Discussion

The presence of the heterogenous subpopulation of malignant phenotypes in tumour tissues has been explained as one of the concepts of CSCs.^28^ However, the origin of CSCs and the niche required for CSC development are still unknown. The CSC generation is the precondition for CSC research, which will help to identify the process of CSC development as well as therapy. Liver cancer is not an exception. Scientists have tried to investigate the niche and molecular mechanisms of CSC development in the liver cancer during the past couple of decades. The development of CSC from iPSCs was described as the conversion of iPSCs into CSC in the presence of CM derived from cancer-derived cell lines.^25^ That was the first report in the light of the conversion of stem cells into CSCs. The chronic inflammation has been reported to have critical roles at different phases of tumour development and might trigger the preliminary mechanism of the tumorigenesis. Since the inflammation may enrich the production of chemokines, cytokines and growth factors, it could induce signal transductions for cell survival and proliferation with chromosomal instability.^29^ This chronic situation might maintain the induction of cellular alteration resulting in malignancy.^30,31^ Thus we thought that the CM from cancer cells, which contains various inflammatory cytokines and chemokines, should be sufficient to induce CSC formation from miPSCs by mimicking chronic inflammation, as we also reported that these pro-inflammatory cytokines and chemokines are activating G protein-coupled receptors and phosphoinositide 3-kinase pathway leading to activate different transcription factors responsible for converting miPSCs into CSCs.^32^

Here, in this study, we demonstrated for the first time that liver CSCs could be generated from iPSCs by culturing in the presence of HCC CM (Huh7) without any genetic manipulation. As a result, we established a protocol to convert iPSCs into liver CSCs. After 4 weeks of culturing miPSCs in the presence of CM, CSCs were induced as miPS-Huh7cm cells, which formed malignant tumours in the liver after 28 days of injection into the liver. Primary cells from the malignant tumour of miPS-Huh7cm cells exhibited the properties similar to liver CSCs, which were defined by self-renewal capacity, differentiation potential and tumorigenicity in vivo.^31^ Converted cells, miPS-Huh7cm cells, were highly tumorigenic and developed malignant tumours after 4 weeks when injected into the liver. These malignant tumours showed significant expression of the markers mostly common to liver cancer such as AFP,^33^ GPC3,^34^ CEA^35^ and CK19.^36^ During the past couple of years, a number of works have identified the membrane-bound markers in liver CSCs. Initially, the characterisation of liver CSCs focussed also in liver cancer on the identification of general CSC markers such as CD24,^37^ CD44^38^ and CD133,^39^ while CK19 was also reported to serve as a relatively specific marker of liver CSCs.^36^ In our study, the malignant tumours developed in the liver showed high and significant expression of CD24, CD44 and CD133 when compared to miPSCs.

The primary cultured cells from all developed tumours showed significant elevation of CSC markers and liver cancer-associated biomarkers.

Taking the results of flow cytometric analysis, it seems that there are different stages of differentiation in vitro from miPSCs up to liver cancer cells with the intermediate stage of liver CSCs with a comparable level of expressing markers. Even in the Nanog-positive stage, the expression levels of liver CSCs could be distinguished by the expression of CEA, GPC3 and AFP.

Our data propose five different stages of stem cells from miPSCs up to liver cancer cells depending on the expression of a panel of markers. Those stages are Nanog+/CEA−/GPC3−/AFP−, Nanog+/CEA+/GPC3−/AFP−, Nanog+/CEA+/GPC3+/AFP−, Nanog+/CEA+/GPC3+/AFP+ and Nanog−/CEA+/GPC3+/AFP+. From these hypothesised stages, an individual marker does not seem to be sufficient to specify liver CSCs. At the same time, it is worthwhile noticing that the undifferentiated stage of CSCs could be distinguished if a panel of markers was used (Supplementary Fig. 6).

Converted cells, miPS-Huh7cm cells, and the tumour-derived primary cells sustained the expression of stemness markers such as Nanog, Sox2, Kif4 and Oct 3/4, which are considered essential for maintaining cell stemness.^40^ These transcription factors related to pluripotency may also contribute to tumorigenesis.^41^ On the other hand, the angiogenesis could be one of the differentiation potentials of CSCs resulting in the differentiation into CD31-positive endothelial cells with expression of VEGF-A and VE-cadherin. Simultaneously, miPS-Huh7cm and the tumour-derived primary cells showed high potential of sphere formation at very limited dilutions as self-renewing character.

Furthermore, metastatic potential should be conceivable to find the expression of MMP2 and MMP9, which are closely associated with the epithelial-to-mesenchymal transition. Collectively, we have successfully demonstrated the preparation of liver CSCs by converting miPSCs in the presence of the CM derived from human liver cancer cell line Huh7 cells.

The liver CSCs converted from miPSCs suffice the definitions of CSCs as well as those of the liver cancer. Not only in the culture containing CM from liver cancer-derived cells but also the tumour formation in the liver tissue enhanced the character of liver CSCs in converted cells. The factors critically related to the liver microenvironment appear to be responsible for the CSC-inducing event observed in the induction of liver CSCs. This process of establishing CSC model^42^ will be useful for understanding the induction process of different CSCs in the future.

## Supplementary information

SUPPLEMENTAL MATERIAL

## Data Availability

All data generated or analysed during this study are included in this published article and its Supplementary File.
