# Supplementary material for: A novel model of liver cancer stem cells developed from induced pluripotent stem cells
Source: Br J Cancer. 2020 Mar 17;122(9):1378–90. doi: 10.1038/s41416-020-0792-z (PMC7188674; doi:10.1038/s41416-020-0792-z)
Supplement: Supplementary file 1 — SUPPLEMENTAL MATERIAL [file 41416_2020_792_MOESM1_ESM.docx]

**Gene expression Met-analysis**

Microarray Data was taken from GEO data base, expression data from the Cancer Cell Lines Encyclopedia, for Huh 7 cell line GSM887149, Hep G2 cell line GSM887079 and PLC cell line was used. Pairwise comparison was conducted using Exatls tool <https://lgsun.irp.nia.nih.gov/exatlas/> between both Huh7 vs HepG2 and Huh7 vs PLC/PFR/5 gene expression data.

**Supplementary Figures**

**
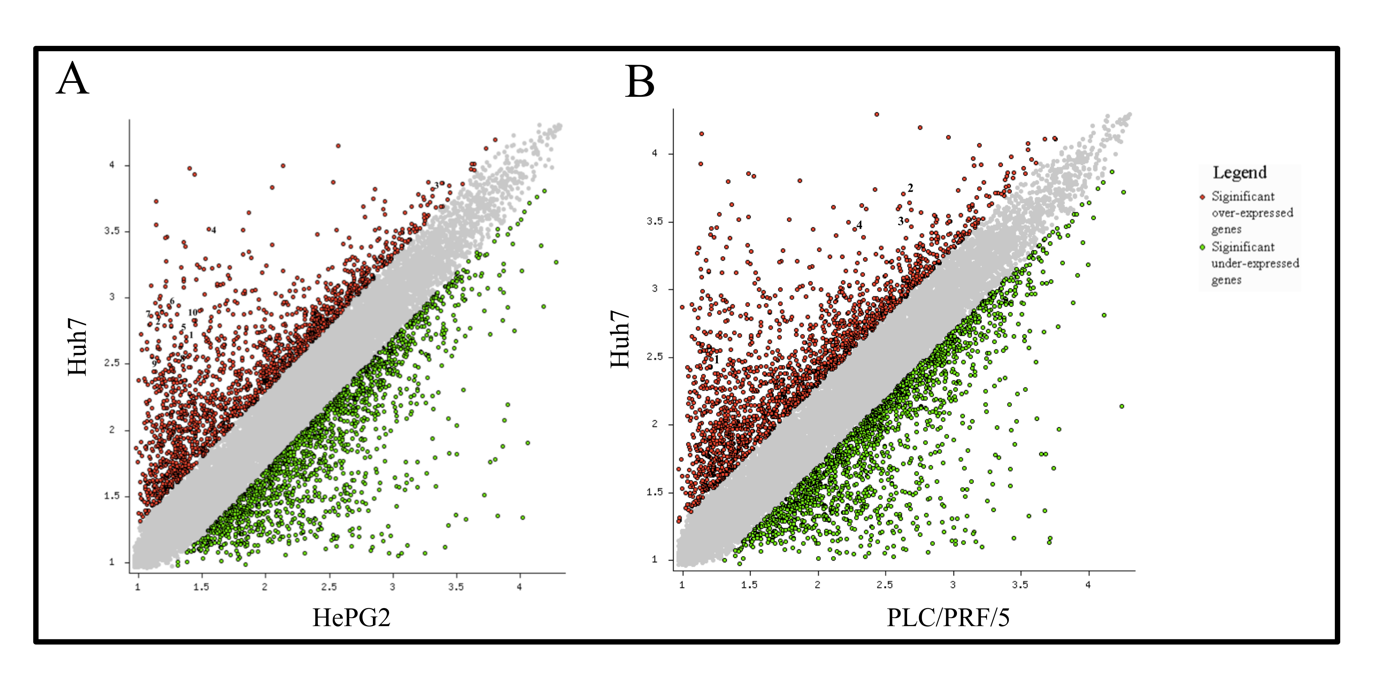
**

**Supplementary Fig. 1** Representative image for scatter plot which showed (A) Over expressed genes in huh 7 compared with hep G2 such as (1) chemokine (C-X-C motif) ligand 1, (2) chemokine (C-X-C motif) ligand 6, (3) ferritin, heavy polypeptide 1, (4) Collagen, type V, alpha 2, (5) glycoprotein hormones, (6) Fibroblast growth factor 19, (7) Tumor necrosis factor, (8) Thrombospondin 4, (9) hyaluronan binding protein 2, (10) RAB31, member RAS oncogene family function cancer

(B) Over expressed genes in Huh7 compared to PLC/PRF/5 such as (1) Thrombospondin 4 (2) glypican 3 (3) Collagen, type V, alpha 2 (4) Bone morphogenetic protein 2

**Supplementary Fig. 2. Representative scheme of the conversion of miPSCs.**

miPSCs can survive in the presence of conditional medium instead of LIF. GFP protein used for Stemness tracking during conversion. Original magnifications 10X.

**Supplementary Fig. 3** Representative image of miPSCs derived teratoma after four weeks of injection as a control which showed a phenotype with various normal germ layers, including squamous epithelium, skeletal muscle, cartilage and benign glandular epithelium.


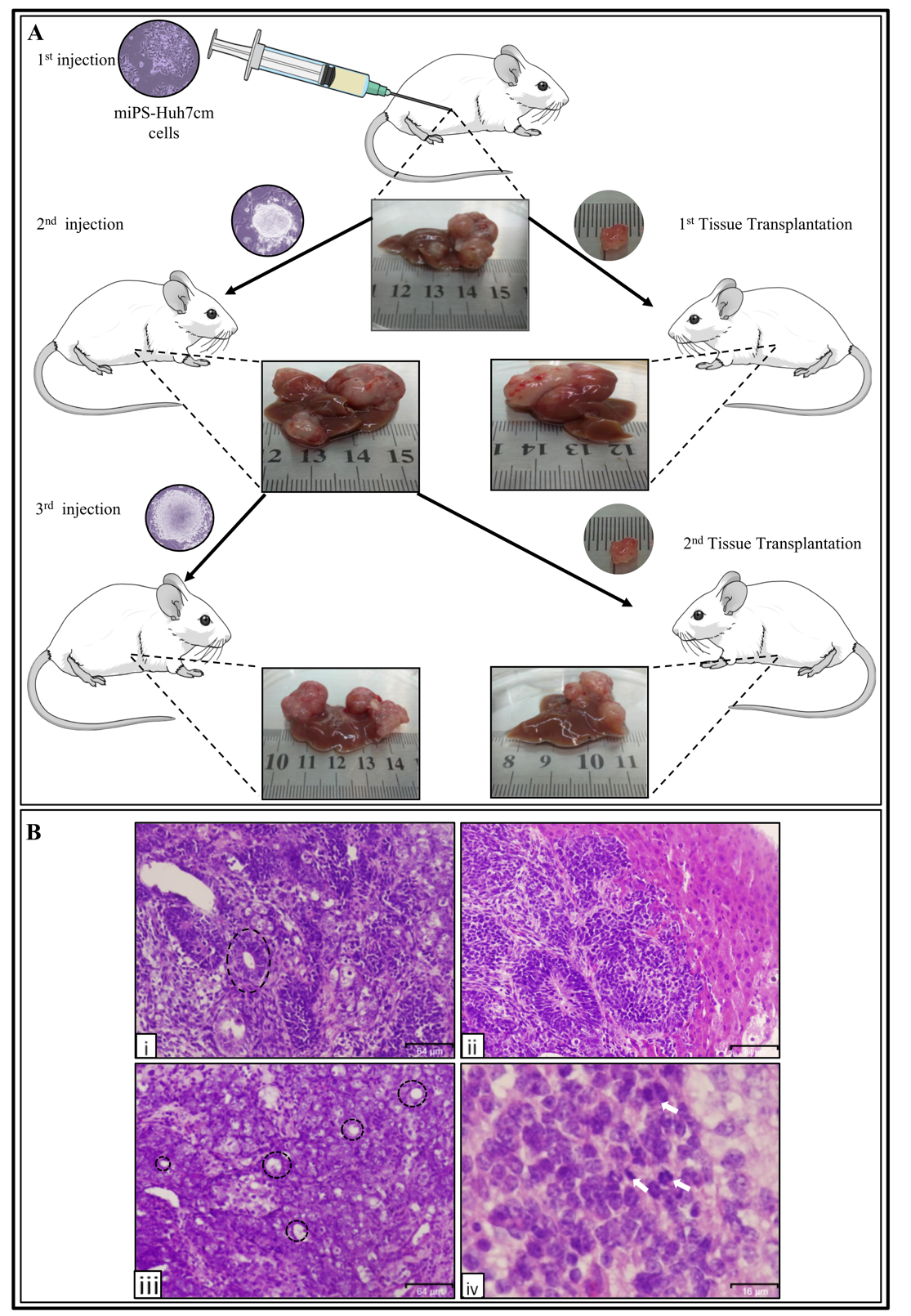


**Supplementary Fig.4: Serial injection for miPS-Huh7cm and primary cultures from derived tumor**

1. Representative images of the serial injection of miPS-Huh7cm cells into the liver and tissue transplantation for the derived tumor
2. H& E staining showed malignant phenotype with glandular epithelial hyperplasia (i), high nuclear to cytoplasmic ratio, and severe nuclear atypia (ii), fatty change (iii), multiple pathological mitotic figures (iv)


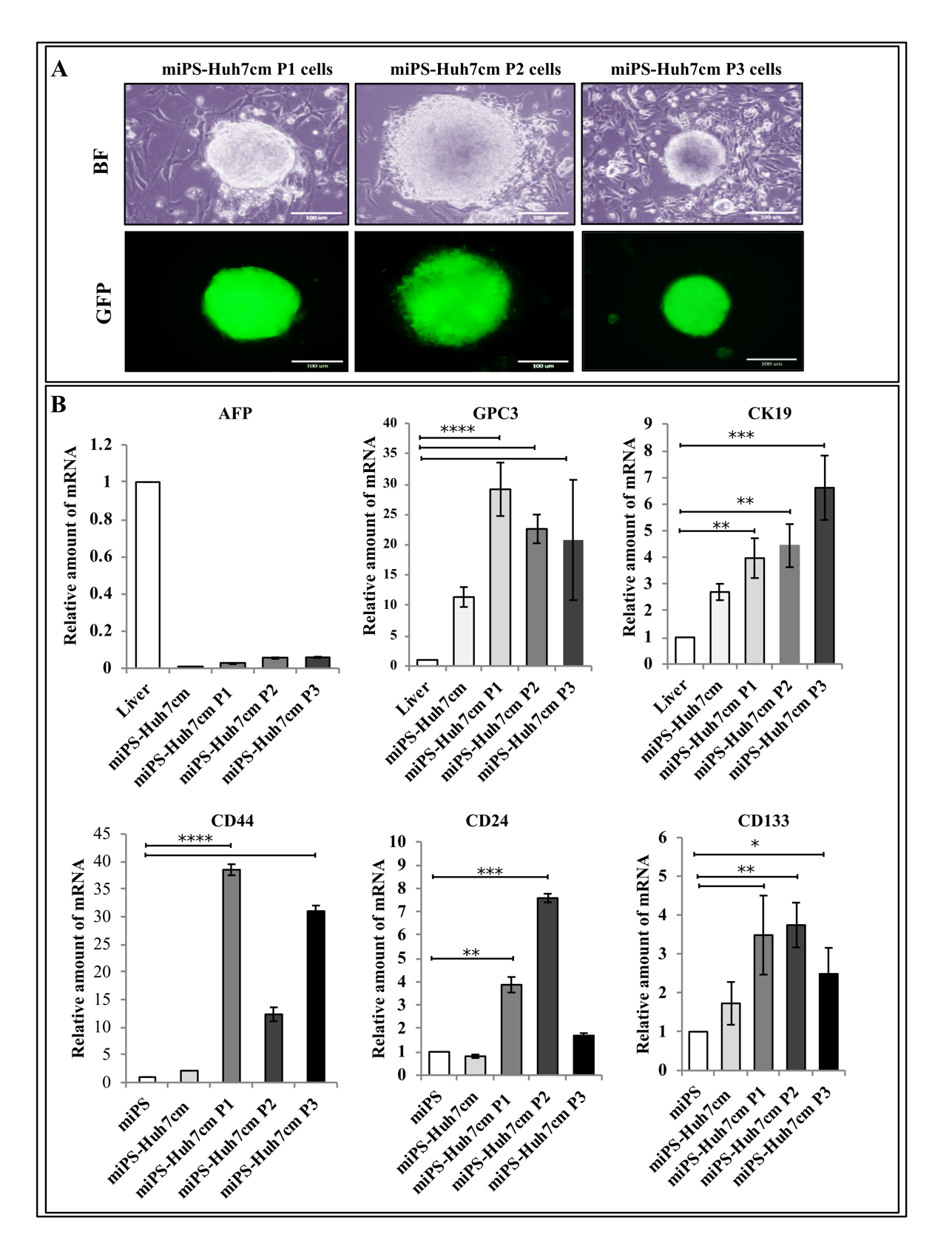


**Supplementary Fig. 5 Characterization of primary cultures cells from different injection**

1. Representative images for adherent cultures of miPS-Huh7cmP1 cells, miPS-Huh7cmP2 cells and miPS-Huh7cmP3 cells. Images shows the differentiated cells and undifferentiated GFP-positive cells, Scale bars represent 100μm
2. RT-qPCR analysis of AFP, GPC3 and CK19 were examined in miPS-Huh7cm and the primary cultures compared to normal adult liver also liver CSC markers CD133, CD44 and CD24 were examined compared to miPS. Gene expression levels were normalized to those of GAPDH


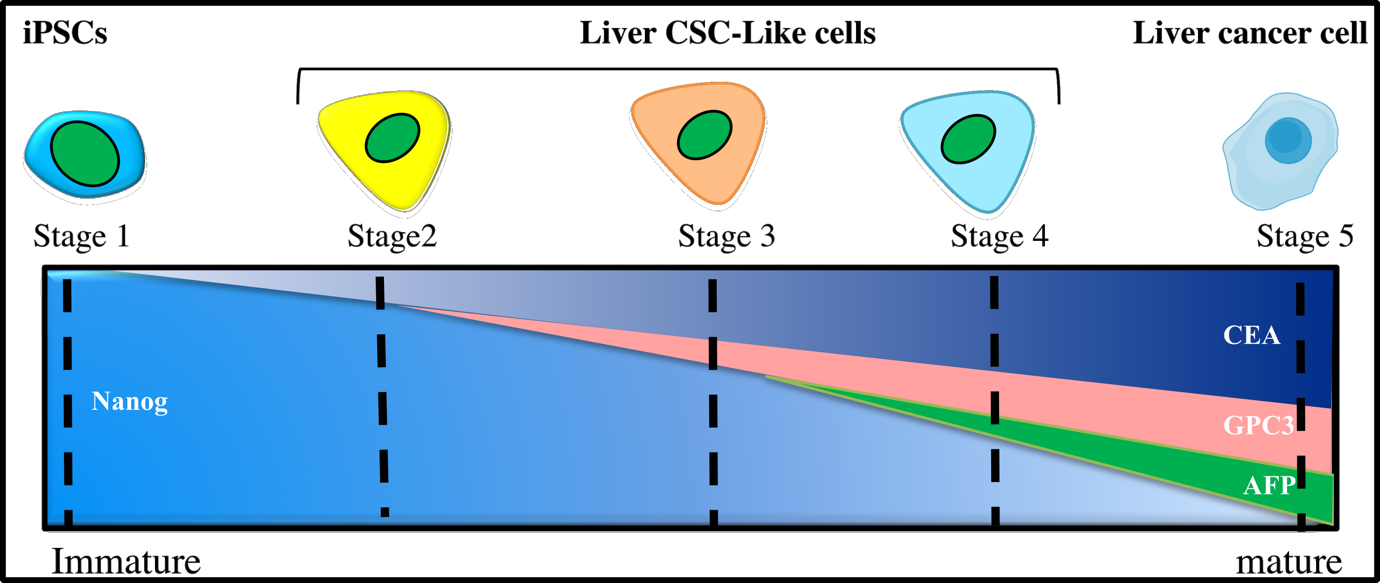


**Supplementary Fig. 6 Stages during miPS differentiation in the presence of conditional medium of hepatocellular carcinoma**

Representative images provide the 5 stages of miPSCs differentiation, Stage 1 (Nanog+/CEA-/GPC3-/AFP-), stage 2 (Nanog+/CEA+/GPC3-/AFP-), stage 3 (Nanog+/CEA+/GPC3+/AFP-), stage 4 (Nanog+/CEA+/GPC3+/AFP+) and stage 5 (Nanog-/CEA+/GPC3+/AFP+).

Supplementary Table. 1 List of Primers Used in the Experiments

Supplementary Table. 2 Antibodies used in the experiments
